# Hypothesis-driven research for G × E interactions: the relationship between oxytocin, parental divorce during adolescence, and depression in young adulthood

**DOI:** 10.3389/fpsyg.2015.01322

**Published:** 2015-09-07

**Authors:** Michael Windle, Sylvie Mrug

**Affiliations:** ^1^Department of Behavioral Sciences and Health Education, Emory University, AtlantaGA, USA; ^2^Department of Psychology, University of Alabama at BirminghamBirmingham, AL, USA

**Keywords:** parental divorce, adolescence, depression, oxytocin, young adulthood

## Abstract

Research in molecular genetics has generally focused on genome-wide association studies (GWAS) and exploratory candidate gene and candidate gene–environment (G × E) studies. In this article it is proposed that hypothesis-driven and biologically informed research provides a complementary approach to GWAS to advance pressing research questions about G × E relations that are of public health relevance. Prior research studies and developmental and evolutionary theory were used to guide hypothesis testing of G × E relationships in this study. The study investigated whether the oxytocin polymorphism, rs53576, moderated the relationship between parental divorce during adolescence and depression symptoms in young adulthood. Oxytocin is a neuropeptide that has been related to the regulation of complex social cognition and behaviors such as empathy, attachment, and nurturance. We hypothesized that the GG polymorphism would be associated with more depressive symptoms following parental divorce, and that this effect would be stronger in females than males. The sample consisted of 340 individuals who participated in a longitudinal study with data used both from adolescence and young adulthood. Findings using prospective follow-up and autoregressive change models supported the hypothesized relationships. Young adult females who had experienced parental divorce during adolescence and had the GG oxytocin genotype reported almost twice as many depressive symptoms relative to young adult females who also experienced parental divorce during adolescence but had the AA or AG genotype. This pattern was not indicated among males. Findings were discussed with regard to how molecular genetic factors in combination with environmental stressors, such parental divorce, framed within a developmental framework may facilitate the future study of G × E relationships in the parental divorce-child adjustment literature and contribute to a prevention science perspective.

## Introduction

An extensive literature on parental divorce has indicated that youth from divorced families more frequently report greater internalizing (e.g., depression, distress, and pain) and externalizing (e.g., delinquency, oppositional behaviors) problems, lower academic performance, and more interpersonal difficulties than children or adolescents from intact families (Cherlin et al., 1998; Booth and Amato, 2001; Lansford, 2009). For example, using long-term prospective data, Hetherington and Kelly (2002) reported that 25% of offspring from divorced families had long-term psychological and social problems relative to only 10% of offspring from families where parents remained together. Similarly, prospective findings by Cherlin et al. (1998) indicated that mental health difficulties among children of divorce relative to children of intact families increased across time when offspring were in their 20 and 30 s. Moderators and mediators of offspring outcomes associated with parental divorce have included factors such as parental conflict preceding the divorce, children’s level of adjustment preceding divorce, and post-divorce parent–child relations (Booth and Amato, 2001; Lansford, 2009). Studies utilizing behavior genetic research designs have supported both genetic and environmental influences on child adjustment following parental divorce (McGue and Lykken, 1992; O’Connor et al., 2000). However, to our knowledge, no study in this area of research has examined how molecular genetic factors may moderate the prospective relationships between parental divorce that occurred for offspring during adolescence, and subsequent depressive symptoms of these offspring during young adulthood.

Research suggests that polymorphisms of the oxytocin receptor gene are involved in the regulation of complex social cognition and behaviors, including empathy, attachment, and nurturance (Bakermans-Kranenburg and van IJzendoorn, 2008; Buchheim et al., 2009; Meyer-Lindenberg et al., 2011). The potential moderating role that a polymorphisms of oxytocin (OXTR), rs53576 (GG), may have in relation to a significant adolescent stressful life event (parental divorce) in terms of depression in adolescence and young adulthood has not been investigated and is the focus of this study.

Beginning in early adolescence, sex differences emerge with regard to depressive symptoms, with girls manifesting significantly higher levels than boys; these sex differences are exacerbated during middle adolescence and are maintained across time with adult women having about twice the rate of major depressive disorders as men (Hankin et al., 1998; Essau et al., 2010). A range of different hypotheses have been forwarded to account for the emergence of sex differences in depression during adolescence (Hankin et al., 1998; Nolen-Hoeksema, 2001; Hyde et al., 2008; Essau et al., 2010). For instance, the onset and development of puberty and associated perceptions of self-esteem and body image differ for boys and girls, with both increased size and masculine characteristics viewed positively by boys but increased size and weight often viewed negatively by girls. These changes associated with pubertal development may contribute to increased discrepancies in depressive symptoms between boys and girls.

Another hypothesis suggests that the adoption and incorporation of gender roles during adolescence, referred to as “gender role intensification” (Hill and Lynch, 1983), may differentially impact boys and girls. Gender role intensification describes male development during this period as being more outwardly focused on autonomy, competitiveness, and self-confidence, whereas female development is more inwardly focused on emotional expressivity, warmth, and care of and support for others. Thus, a major disruptive event such as parental divorce may differentially impact age-appropriate developmental role performance for boys and girls, thereby contributing to higher levels of depression among females. Findings by Mustonen et al. (2011) indicated that parental divorce during adolescence was associated prospectively with poorer intimate relationship quality in adulthood (16-year follow-up) among daughters than sons.

Yet another perspective on gender differences in depression is provided by an evolutionary life history framework for psychopathology (Del Giudice, 2014). Prior research has suggested that men are more susceptible to depression due to status loss, whereas women are more susceptible due to reduced social support (Kendler et al., 2005; La Greca et al., 2009). Building upon these findings, Del Giudice (2014) has incorporated stress-related components into his model of depression, in addition to affective reactivity, and has emphasized the need to investigate both affective reactivity and stress reactivity in relation to slow or fast life history strategies. Furthermore, it is proposed that across development men develop more unemotional responses characterized by a more hypo-responsive stress response system whereas females may develop more hyper-responsive stress systems. The occurrence of a major stressor such as parental divorce makes the environment less stable and more chaotic, whereby social support and stable intimate relationships can decrease rapidly. Because females have more hyper-responsive stress response systems than males, and are differentially affected by disrupted social environmental factors (e.g., social support from parents and family members), the expectation would be that females would respond more adversely to parental divorce than males through the expression of more depressive symptoms.

There is increasing evidence that sub-threshold levels of depressive symptoms (i.e., expressing some depressive symptoms but not a sufficient number to meet clinical diagnostic criteria) in adolescence are clinically significant in predicting subsequent mental health problems in young adulthood (Lewinsohn et al., 2000; Cuijpers and Smit, 2004). For example, findings by Gotlib et al. (1995) indicated that adolescents with high scores on the Center for Epidemiologic Studies Depression Scale (CES-D; Radloff, 1977) who did not meet formal clinical diagnostic criteria for a depressive disorder did not differ from adolescents meeting these criteria on several measures of psychopathology and psychosocial dysfunction. Furthermore, several two-wave prospective studies indicated that elevated, but subclinical depressive symptoms in adolescence were a risk factor for the development of adult depressed mood (Kandel and Davies, 1986) and adult depressive disorder (Rao et al., 1999; Cuijpers and Smit, 2004). The implication of these findings is that high levels of depressive symptoms among adolescents, as reported on self-report measures such as the CES-D, pose a serious public health problem among adolescents in transition to young adulthood in that they may predict aggravated mental health problems and disorders through the lifespan.

Oxytocin is a neuropeptide that impacts a range of response systems including some related to reproduction (uterine contraction during childbirth) and early childhood care (e.g., milk ejection during breastfeeding) and others associated with a range of social cognitive and social affiliative behaviors (Bakermans-Kranenburg and van IJzendoorn, 2008; Costa et al., 2009; Meyer-Lindenberg et al., 2011). The rs53576 SNP is located on chromosome 3p25 and has unknown biological functionality in the third intron of the larger oxytocin gene (OXTR). Research findings for rs53576 and social affiliative behaviors have been consistent in demonstrating significant statistical associations, but the identified risk alleles of those associations have not always been consistent. For example, Rodrigues et al. (2009) reported that A allele carriers had lower empathy and lower positive affect, and manifested greater physiological stress reactivity. Similarly, Bakermans-Kranenburg and van IJzendoorn (2008) reported that carriers of the AA/AG genotypes demonstrated lower levels of maternal responsiveness to toddlers. By contrast, Costa et al. (2009) found that GG allele carriers had higher levels of separation anxiety in adulthood, including a higher need for social approval and lower confidence with respect to having a secure attachment. Norman et al. (2012) also provided evidence that GG carriers manifested higher levels of sympathetic reactivity to psychological stress.

Although these sets of findings are in conflict, there is emerging evidence that inconsistencies may be attributable to genotype × environment interactions. Chen et al. (2011) used a laboratory design to investigate the possibility that rs53576 might interact with stress-protective effects of social support. They found that social support prior to a laboratory-based stressor was associated with lower cortisol and subjective stress responses for G allele carriers, but not for AA carriers. Hence, there was a genotype by stress (environment) interaction effect. Similarly, Sturge-Apple et al. (2012) reported a statistically significant interaction for oxytocin × interparental conflict on maternal sensitivity with toddlers. Across low and high levels of interparental conflict, AA/AG carriers did not differ in maternal sensitivity to their toddlers; however, GG carriers demonstrated plasticity across levels of interparental conflict with low levels of conflict associated with high sensitivity and then sensitivity decreasing significantly as interparental conflict increased. Bradley et al. (2011) reported an interaction between childhood maltreatment and the rs53576 SNP in that GG genotype carriers were at increased risk for emotional dysregulation and a disorganized attachment style relative to AA and AG carriers. These studies suggest that GG carriers may be most sensitive to positive and negative environmental influences. Collectively, these findings suggest the potential benefits of considering gene × environment interactions when examining the role of oxytocin and stress on social affiliative behaviors. Furthermore, some findings suggest that sex may further moderate the strength of associations between oxytocin, stress, and social affiliative behaviors (Tost et al., 2010).

In this study, we used literature on sex differences in depression and differential responses to stressors, as well as literature on sex role socialization (e.g., gender role intensification) and evolutionary theory, as a basis to examine hypotheses about sex differences in the conditional (G × E interactional) relationship between parental divorce occurring during adolescence and the oxytocin polymorphism rs53576. Specifically, we hypothesized that divorce occurring during adolescence would interact with the rs53576 polymorphism to prospectively predict depressive symptoms among young adult females but not young adult males. To the extent that parental divorce during adolescence contributes to a greater disruption of the inwardly focused gender role developmental tasks (e.g., emotional expressivity, warmth, and care of and support for others) of females relative to the outward-focused male tasks, and reduces the quality of the social environment that is evolutionarily more important to females, we would expect greater increases in depressive symptoms in females. Additionally, the GG oxytocin genotype should make females even more susceptible to the negative effects of parental divorce. Thus, females with the GG genotype who also had experienced divorce during adolescence would be hypothesized to have significantly higher depression scores in young adulthood than females not homozygous for GG and all males.

## Materials and Methods

### Participants

The data used in this report were collected as part of a larger, multi-wave panel design study focused on risk and protective factors and adolescent and young adult substance use and mental health. We refer to the study by the acronym LAT, which stands for Lives across Time: A Longitudinal Study of Adolescent and Adult Development (for details, see Windle et al., 2005). Initially, data were collected from adolescents in their high school setting and the overall student participation rate was 76%. The average age of the respondents at the first occasion of measurement was 15.54 years (*SD* = 0.66) and 98% were white. Sample retention across the first four waves of measurement was uniformly high, in excess of 90%.

In this study we used data from 11th and 12th grade participants in all four adolescent waves (with 6-month intervals between assessments) and their follow-up young adult wave (Wave 5) approximately 6-years later when the average age of the young adults was 23.5 years. In-school surveys were completed during adolescence and individual interviews were conducted in young adulthood. For the current study, supplemental funding was provided by NIAAA to collect and analyze DNA for a subsample of the LAT (there were insufficient funds and an abbreviated time window to collect data from all participants). The subsample selected consisted of 340 participants with priority of selection given to subjects who participated during both adolescence and young adulthood to maximize the prospects of testing developmental gene–environment (G × E) prospective relationships. The 340 individuals who participated did not differ significantly from those who did not participate on key variables (e.g., family income, adolescent alcohol use, adolescent depression, and grade-point average).

### Procedure

During the adolescent phase, subsequent to receiving informed consent both from a parent and the target adolescent, a trained survey research team administered the survey to adolescents in large groups (e.g., 40–50 students) in their high school setting at each wave. The survey took about 45–50 min to complete and students received $10 for their participation. The study was approved by the University at Buffalo IRB and confidentiality was further assured with a U.S. Department of Health and Human Services Certificate of Confidentiality. The young adulthood interview at Wave 5 was conducted via one-on-one interviews either in the participants’ homes or at the host institute of the investigators. Adults were paid $40 to complete an interview that lasted approximately 2 h. Computer-assisted personal interviews were used to collect the survey data.

Saliva samples were provided from a subset of the sample via a mail data collection protocol using Oragene DNA kits (Genetek; Calgary, AB, Canada). Participants were instructed to rinse their mouths with tap water and then deposit 4 ml of saliva in the Oragene sample vial. The vial was sealed, inverted, and shipped via courier first to the location of the study’s primary investigator and then to the Georgia Genomics Facility (GGF) at the University of Georgia (http://dna.uga.edu) where DNA was extracted from saliva samples according to the manufacturer’s specifications.

### Measures

#### Sociodemographic Variables

In their individual interviews and the completion of mail surveys (during the adolescent phase of the study), participating parents were asked about their age, number of years of education completed, family income, and other demographics (e.g., marital and occupational status). Family income (during the target’s adolescence) and highest level of education by either parent were used as covariates in data analyses.

#### Parental Divorce

Parental divorce or separation was assessed by an item within a list of 31 undesirable life events that was constructed by modifying the brief (24-item) form of the Adolescent Life Change Event Scale (ALCES; Forman et al., 1983) as detailed by Windle (1987). At each of the four waves of measurement that occurred during adolescence, adolescents were requested to report whether each of the life events occurred during the previous 6 months. The event was worded as “parental divorce or separation” with a Yes/No response format. Parental divorce was coded as a dichotomous variable indicating whether parental divorce or separation was endorsed at any of the four occasions. For this sample, 8.8% of the adolescents reported parental divorce across the 2-year interval. Parental divorce during adolescence as reported by adolescents was used in this study because of the proposed (adolescent) developmental significance in reference to the proposed hypotheses. The possible impact of parental divorce on child outcomes may vary contingent on age, sex, and a host of other variables that were beyond the scope of this study.

#### Depressive Symptoms

At Waves 4 and 5, depressive symptoms were assessed using the CES-D (Radloff, 1977). The CES-D consists of 20 self-report items and provides a unitary measure of current depressive symptoms, with an emphasis on the affective component, depressed mood. Participants were asked to indicate how many days during the past week they had experienced the emotions or behaviors indicated in each of the items. The response options for these items ranged from “0 = *Rarely or none of the time*” to “3 = *Most or all of the time*.” The internal consistency estimate for the CES-D in this sample was 0.90 at Wave 4 and 0.91 at Wave 5.

#### Oxytocin (OXTR)

DNA was extracted from saliva samples according to the manufacturer’s specifications at the Georgia Genomics Facility (GGF) at the University of Georgia (http://dna.uga.edu). Genotyping was conducted by a technician blind to other data from the research project. High throughput genotyping was completed for single nucleotide polymorphisms (SNPs) on BeadXpress using the GoldenGate Genotyping Assay for VeraCode from Illumina. A customized set of SNPs was provided to Illumina by the investigator and Illumina provided the final oligonucleotides sequences to be used in the GoldenGate assay at the GGF. Quality control data procedures for the SNPs included a genotype call rate of 98% and subject filtering per SNP call rates equal-to-or-greater-than 95%. Exclusion of SNPs also occurred if the minor allele frequency rate (MAF) was less than 1%, there was significant departure from Hardy–Weinberg equilibrium at *p* < 10^-4^, or outliers occurred (using a criterion of more than 5 SD). The OXTR rs53576 polymorphism was assessed as part of the SNP panel and met the criteria described above. The genotype distribution of OXTR for AA was 10.0% (*n* = 34), AG 41.2% (*n* = 140), and GG 48.8% (*n* = 166). Consistent with standard scoring for this SNP (e.g., Sturge-Apple et al., 2012), AA and AG genotypes were combined and compared with the GG group.

### Data Analyses

Preliminary analyses examined frequency distributions of variables to identify outliers and influential data points, as well as non-normal distributions. For example, with respect to the latter, skewness for the depression scale at Wave 4 was –0.35 and at Wave 5 was 1.29. These values do not deviate largely from a symmetric distribution and the use of transformed variables for the Wave 5 depression score did not alter substantive findings from the use of raw scores. Also, no outliers or influential data points were observed for depression for children from divorced and non-divorced families. A linear regression model was used to specify two different prospective models to predict Wave 5, young adult depression. The first specified model included Wave 5 depression as the dependent variable and the predictor variables consisted of the two covariates of parental education and family income, the three “main effects” of Sex, OXTR (rs53576), and parental divorce status; the three 2-way interactions of Sex × parental divorce status, sex × OXTR, and OXTR × parental divorce status; and the three-way interaction of Sex × parental divorce status × OXTR. Because of the smaller sample size to evaluate G × E interactions, bootstrapping was applied using the Mersenne twister (Matsumoto and Nishimura, 1998) and 1,000 bootstrap samples.

A second, highly similar prospective model was used to predict Wave 5, young adult depression, but depressive symptoms at Wave 4 were included in the specified model as an additional covariate to control for levels of adolescent depression. By including Wave 4 depression, a subtle but important distinction was made in that the model now predicted *change* in depression from adolescence to young adulthood rather than just prospectively predicting young adult levels of depression. This distinction is important because some studies have indicated high levels of continuity between depression in adolescence and young adulthood (Kandel and Davies, 1986; Cuijpers and Smit, 2004); without controlling for depression in adolescence, it would be difficult to know if the hypothesized G × E interaction existed in adolescence to impact depression and was then carried forward into young adulthood, or if it appeared to impact *changes* in depression from adolescence to young adulthood. Support for either of the two specified models would be valuable to the literature, but the interpretation would differ if the first model supported the G × E interaction but the second model did not. Hence, by specifying and evaluating both models a more rigorous evaluation of the hypothesized G × E interaction was facilitated. The statistical significance level of each model was provided, as well as the statistical significance of individual parameters and an overall model estimate of the variance accounted for (i.e., an adjusted *R*^2^-value).

## Results

Zero-order correlations and descriptive statistics for the sample are provided in **Table 1**. The sex distribution was females (*n* = 201; 59.1%) and males (*n* = 139; 40.9%), and 30 (8.8%) participants reported parental divorce status during adolescence. In bivariate comparisons via contingency tables, the distribution of the OXTR genotype did not differ significantly across sex groups [χ^2^ (1) = 0.22, *p* = 0.66] or divorce status [χ*^2^* (1) = 1.95, *p* = 0.16], and sex group did not differ significantly across divorce status [χ^2^ (1) = 0.11, *p* = 0.94].

**Table 1:** Correlations, means, and SD of demographic variables, divorce status, oxytocin, and depression (*N* = 340).

Variable	1	2	3	4	5	6	7
(1) Parent education	1.00						
(2) Family income	0.29	1.00					
(3) Gender (1 = M; 2 = F)	0.01	0.02	1.00				
(4) Divorce (0 = no; 1 = yes)	–0.01	–0.02	0.01	1.00			
(5) Oxytocin (0 = AA/AG; 1 = GG)	0.02	–0.01	–0.03	–0.08	1.00		
(6) W4 depression	0.12	–0.06	0.06	0.15	–0-.04	1.00	
(7) W5 depression	–0.06	–0.03	0.01	0.06	–0.02	0.33	1.00
*M*	2.88	6.42	1.59	0.09	0.49	15.02	10.29
*SD*	0.82	1.87	0.49	0.28	0.50	10.15	7.87

**FIGURE 1:**
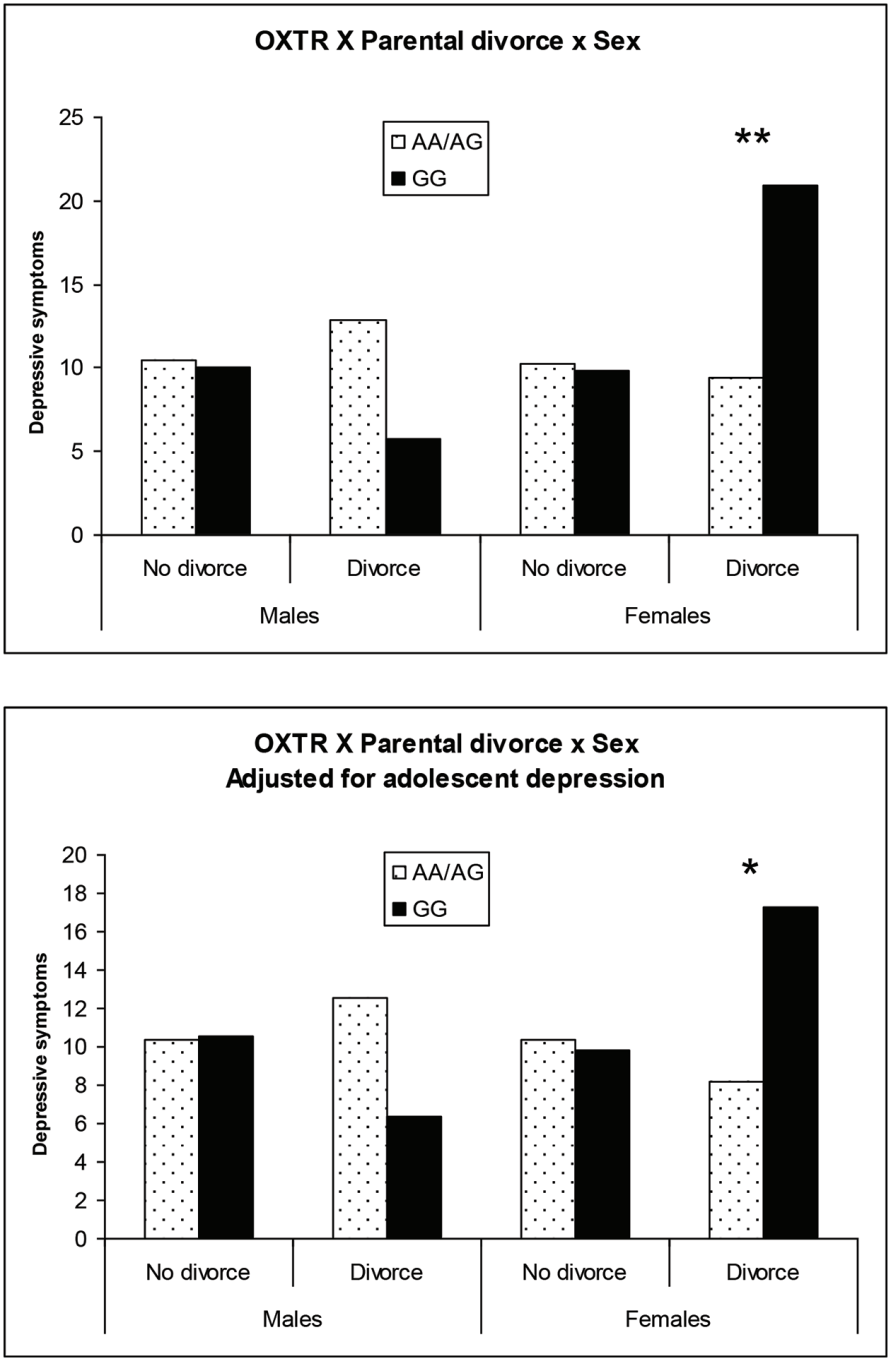
**Plot of significant OXTR × Sex × Parental divorce during adolescence interaction on young adult depression for males and females without and with adjustment for adolescent depression.** ^∗^*p* < 0.05; ^∗∗^*p* < 0.01.

The findings of the first regression model are summarized in **Table 2**. The overall model was statistically significant at *p* < 0.10 and young adult depression was significantly predicted by the hypothesized three-way interaction of Sex × OXTR × Parental divorce status. To facilitate the interpretation of this three-way interaction, the adjusted means of subgroups defined by sex, parental divorce status and OXTR genotype were plotted (**Figure 1**, top). Among males who experienced parental divorce, the proposed risk allele (GG) was associated with slightly lower depressive symptoms compared to the AA/AG genotype, but the difference was not significant [Cohen’s *d* = –0.17; *t*(330) = –1.54, *p =* 0.12]. By contrast, for females who experienced parental divorce, the proposed risk allele (GG) was related to significantly elevated CES-D depression scores from 9 to 21 [Cohen’s *d* = 0.32; *t*(330) = 2.95, *p <* 0.01]. No other differences between the subgroups emerged. These findings support the hypothesized three-way interaction in the prediction of depressive symptoms in young adulthood.

**Table 2:** Bootstrapped regression model findings predicting depression in young adulthood (Wave 5)^1^.

Source	B	Bias	*SE*	Significance
Intercept	11.70	–0.04	2.36	0.001
Parental education	–0.30	0.01	0.50	0.552
Family income	–0.02	–0.01	0.19	0.931
Sex (Female)	–0.21	0.07	1.18	0.859
Oxytocin (OXTR; GG)	–0.43	0.20	3.01	0.905
Divorce status	5.57	0.24	5.46	0.290
Sex^∗^Divorce	–3.22	–0.20	3.50	0.331
Sex^∗^OXTR	–0.03	–0.15	1.81	0.989
OXTR^∗^Divorce	–25.19	–0.10	9.87	0.005
Sex^∗^OXTR^∗^Divorce	18.60	0.19	7.19	0.006

*^1^The *F*-statistic was 1.69, *p* = 0.089 and the adjusted *R*^*2*^-value was 0.044.*

The findings of the second regression model that included Wave 4 depression as a covariate, are summarized in **Table 3**. The overall model was statistically significant and again young adult depression was significantly predicted by the hypothesized three-way interaction of Sex × OXTR × Parental divorce status. Adjusted means for all subgroups are depicted in **Figure 1** (bottom). Findings for this second model largely paralleled those of the first model. Among males who experienced parental divorce, the proposed risk allele (GG) was associated with lower depression scores, but the difference was not significant [Cohen’s *d* = –0.15; *t*(329) = –1.36, *p =* 0.18]. By contrast, the proposed risk allele (GG) was linked with higher levels of depression among females who had experienced parental divorce compared to the AA/AG allele carriers, with mean CESD-depression scores of 17 vs. 8 [Cohen’s *d* = 0.26; *t*(330) = 2.33, *p <* 0.05]. No other subgroups differed. These findings support the hypothesized three-way interaction in the prediction of change in depression from adolescence to young adulthood, although the effect sizes were of small magnitude.

**Table 3:** Bootstrapped regression model findings predicting depression in young adulthood (Wave 5) controlling for adolescent depression (Wave 4)^1^.

Source	B	Bias	*SE*	Significance
Intercept	8.21	–0.02	2.26	0.002
Parental education	–0.80	0.02	0.49	0.098
Family income	0.12	–0.01	0.20	0.540
Sex (Female)	–0.02	0.03	1.10	0.990
Oxytocin (OXTR; GG)	0.88	0.18	2.93	0.777
Divorce status	6.48	0.18	5.07	0.188
W4 depression	0.25	0.01	0.05	0.001
Sex^∗^Divorce	–4.32	–0.17	3.25	0.169
Sex^∗^OXTR	–0.71	–0.13	1.75	0.685
OXTR^∗^Divorce	–22.34	–0.11	8.58	0.004
Sex^∗^OXTR^∗^Divorce	–15.99	0.18	6.61	0.010

*^1^The *F*-statistic was 5.27, *p* < 0.000 and the adjusted *R*^*2*^-value was 0.112.*

## Discussion

The findings of this study supported the hypothesized three-way interaction between sex, OXTR, and a significant adolescent stressor—parental divorce. Females with the GG risk allele manifested significant increases in depressive symptoms in young adulthood if parental divorce had occurred for them during adolescence; this relationship was not indicated for males. The findings were robust across both the prospective follow-up design in which adolescent depression was not controlled, as well as a longitudinal autoregressive change model in which adolescent depression was controlled, thereby supporting the interpretation that the three-way interaction predicted change in depressive symptoms from adolescence to young adulthood.

These findings contribute to the literature on moderators of the relationships between parental divorce and offspring depression in two ways (Booth and Amato, 2001; Lansford, 2009). First, they identify a molecular genetic factor, the OXTR polymorphism rs53576, as a statistically significant modifier of the parental divorce-offspring adjustment relationship, thereby opening-up new avenues of research that include genetic markers along with other pre- and post-divorce psychological and social factors. Second, the findings highlight potential advantages of embedding possible G × E relationships for the impact of parental divorce within a developmental framework, in this instance combining developmental studies of significant events/process (e.g., sex role development, sex differences in depression) in adolescence with findings of consilience in genetics, infrahuman studies, and cognitive neuroscience (Bakermans-Kranenburg and van IJzendoorn, 2008; Buchheim et al., 2009; Meyer-Lindenberg et al., 2011). Further investigation of this issue using the evolutionary perspective of Del Giudice (2014) are also merited, as it provides a cross-level, integrative approach.

The specific findings of this study for OXTR are most consistent with the findings of Costa et al. (2009), Bradley et al. (2011), and Sturge-Apple et al. (2012). We reported that OXTR interacted with a family stressor, parental divorce, during adolescence, to predict depression in young adulthood (differentially for males and females). Sturge-Apple et al. (2012) reported that OXTR interacted with a family stressor, interparental conflict, and maternal sensitivity to toddlers as their outcome, and a similar G × E interaction was supported. Likewise, Costa et al. (2009) reported that the GG allele combination was associated with the highest levels of separation anxiety and insecure attachments among adults with depression. Our study does not resolve the mixed findings that have been reported in the OXT-social affiliative behaviors literature (Bakermans-Kranenburg and van IJzendoorn, 2008; Costa et al., 2009), but does provide additional support for considering G × E interactions, possible sex differences (Tost et al., 2010), and expanding the age range for developmental studies beyond infancy and childhood.

A major limitation of candidate gene studies has been the failure of replication, due in part, to underpowered sample sizes and to a failure to correct for multiple hypothesis testing (Duncan and Keller, 2011; Duncan et al., 2014; Manuck and McCaffery, 2014). An underpowered sample size is a limitation of this study, although the sample sizes and cross-validation samples of tens of thousands recommended for GWAS are highly improbable for research designs (e.g., clinical trials, neuroimaging studies, pharmacogenetic studies) and complex phenotypes of interest in psychology, psychiatry, and the behavioral sciences. Furthermore, it often is challenging to replicate developmental G × E studies or to conduct G × E meta-analyses because studies would require similar samples, with similar measures, and similar time-points (or waves of assessment). Nevertheless, replication studies are clearly needed to confirm and extend the findings of this, and other behavioral science studies. The limitations of candidate gene studies, such as the current one, can also be reduced by using extant empirical findings and theories, including GWAS findings and developmental and evolutionary theories, to guide hypotheses formation and to form polygenic indexes. Issues related to small sample size, relative to other sampling strategies (e.g., GWAS), can also be attenuated by selecting a specific genotype or small set of genotypes (e.g., polygenic indexes) rather than using exploratory data-mining procedures that require stringent corrections to the nominal alpha level for statistical testing. This study also has other limitations such as sample restrictions with respect to the representation of diverse ethnic groups and to a broader socioeconomic range. It also does not contain variables that would facilitate the evaluation of the intervening mediators and mechanisms that may account for the obtained relationships, and other risk factors for young adult depression were not included in the models. Despite these limitations, the findings do provide insight into how G × E relationships, embedded within a well-supported research literature, can advance our understanding of parental divorce and young adult depression.

## Author Contributions

Both authors (MW and SM) made substantial contributions to the conception and design of this article. MW conducted most of the data analyses and both authors contributed to the interpretation of findings. Both authors participated in drafting and revising the work for intellectual content, and both provided final approval of the version to be published. The authors also agreed to be accountable for all aspects of the work in ensuring that questions related to the accuracy or integrity of any part of the work are appropriately investigated and resolved.

## Conflict of Interest Statement

The authors declare that the research was conducted in the absence of any commercial or financial relationships that could be construed as a potential conflict of interest.
